# An evidence-to-claim framework for cGAS–STING and AIM2 in atherosclerosis

**DOI:** 10.3389/fcell.2026.1999070

**Published:** 2026-10-22

**Authors:** Lin Lu, Hui Lin, Peng Zhang, Yun Li, Jie Zhang

**Affiliations:** 1 Department of Medical Research Center, Shaoxing People’s Hospital, Shaoxing, China; 2 School of Medicine, Shaoxing University, Shaoxing, China; 3 Department of Cardiology, Shaoxing People’s Hospital, Shaoxing, China

**Keywords:** AIM2 inflammasome, atherosclerosis, cGAS-STING, cytosolic self-DNA sensing, mitochondrial DNA, vascular cell compartments

## Abstract

Cytosolic self-DNA sensing has been implicated in atherosclerosis, but similar pathway readouts are often interpreted as equivalent across cell compartments and disease stages. We evaluate DNA identity and localization, node causality, cellular lineage, and endpoint relevance before assigning a biological or therapeutic inference. Within the evidence considered in this focused narrative review, the STING signaling adaptor is most directly linked to disturbed-flow-associated endothelial dysfunction. In macrophage and hematopoietic compartments, STING-node dependence supports chronic inflammatory amplification. Vascular smooth muscle cell studies support a DNA-damage-associated STING-node stress program, whereas the requirement for cell-autonomous cGAS signaling and structural causality remains unresolved. AIM2 is a parallel, context- and stage-dependent axis in JAK2V617F clonal hematopoiesis, selected advanced-plaque models, and acute post-stroke neutrophil extracellular trap (NET)-DNA exposure. These settings map to distinct endpoints and should not be treated as interchangeable therapeutic indications. This focused narrative review applies an author-developed heuristic framework rather than a validated evidence-grading instrument; none of the studies considered fulfilled the complete same-model L2/N2/C2/E2 benchmark.

## 1 Introduction

Atherosclerosis arises from lipid retention, endothelial dysfunction, immune activation, maladaptive repair, and arterial-wall remodeling (Libby, 2021). Oxidative stress, disturbed flow, hyperlipidemia, inflammation, and mitochondrial dysfunction can damage nuclear or mitochondrial DNA (Cervelli et al., 2012). DNA damage alone is not an innate immune signal. A pathogenic DNA-sensing model requires self-DNA to escape its normal compartment, engage a defined sensor, initiate a signaling program, and alter a relevant vascular or lesion phenotype (Ablasser et al., 2013a; Ding et al., 2024; Gluck et al., 2017; Li and Chen, 2018).

Recent reviews commonly present cyclic GMP-AMP synthase (cGAS) and the stimulator of interferon genes (STING) signaling adaptor as one pathway narrative (Marian, 2025; Wang et al., 2025; Zhang et al., 2024). This review is organized around a central premise: the same pathway marker does not support the same claim across cell types, disease stages, and endpoints. It asks which cell- and stage-specific claim is supported by DNA localization, sensor perturbation, lineage, and a matched vascular or plaque endpoint. Reduced pathway transcripts indicate target engagement, not vascular benefit. The 2025 review by Zhang et al. summarized mtDNA damage, release, immune signaling, biomarker potential, and therapeutic perspectives in atherosclerosis; the present review instead asks when such observations are sufficient to support a same-cell DNA-to-sensor causal inference (Zhang et al., 2025).

The same molecular signal can arise in disturbed-flow endothelium, macrophages with defective clearance, or VSMCs under genotoxic stress. These settings have distinct physiological consequences: endothelial activation is relevant to initiation, macrophage inflammatory amplification to progression, and VSMC or matrix changes to structural risk. Interventions should therefore be judged against the function they are intended to improve, using the stage criteria in Section 2.

Within the evidence considered in this focused narrative review, four conclusions organize this review: endothelial STING is a stress-responsive early-dysfunction model, most directly in disturbed flow; hematopoietic/STING signaling can amplify chronic plaque inflammation; VSMC DNA-sensing stress signaling is a CKD-associated cellular program with unproven structural causality; and AIM2 is a parallel axis in clonal hematopoiesis and acute NET-DNA exposure. These models imply different therapeutic hypotheses and are not interchangeable pathway readouts. Their falsifiable predictions are listed in Supplementary Table S1.

cGAS and absent in melanoma 2 (AIM2) are DNA sensors whose ligand-recognition events considered here occur when double-stranded self-DNA is mislocalized or otherwise becomes accessible in the cytosolic compartment. cGAS is not exclusively cytosolic: nuclear cGAS is normally tethered to nucleosomes, whose acidic patch restrains cGAS dimerization and activation by chromatinized self-DNA (Kujirai et al., 2020). When accessible DNA engages cGAS productively, it generates 2′3′-cyclic GMP-AMP (2′3′-cGAMP), which activates STING, an endoplasmic-reticulum-resident signaling adaptor, and downstream TANK-binding kinase 1 (TBK1), interferon regulatory factor 3 (IRF3), and nuclear factor-κB (NF-κB) signaling (Ablasser et al., 2013a; Li and Chen, 2018). AIM2 binds cytosolic double-stranded DNA, recruits the apoptosis-associated speck-like protein containing a CARD (ASC), and activates caspase-1, IL-1β/IL-18 maturation, and potentially gasdermin-D-dependent pyroptosis (Fernandes-Alnemri et al., 2009). The two pathways may respond to overlapping DNA sources but are not obligatory intermediates of one another.

Previous reviews have primarily summarized cGAS–STING activation, downstream signaling, and disease associations (Marian, 2025; Wang et al., 2025; Zhang et al., 2024). This review instead specifies the evidentiary conditions required to move from a pathway observation to a cell-specific, disease-stage-matched mechanistic or therapeutic inference: a defined and localized DNA source, sensor-node causality, lineage restriction, and a relevant vascular or plaque endpoint. Accordingly, equivalent STING-pathway readouts in endothelium, macrophages, and VSMCs cannot be assumed to support an equivalent therapeutic strategy. Thus, whereas prior reviews generally use the pathway as the primary unit of synthesis, the present review uses the defined DNA source–sensor node–cell lineage–stage-matched endpoint as its unit of assessment.

### 1.1 Scope and literature approach

This focused narrative review considered English-language literature retrieved from PubMed and Web of Science from January 2009 through 14 September 2026 and complemented this reading with cited-reference screening. Searches combined atherosclerosis and related vascular contexts with cytosolic DNA-sensing, cell-compartment, and endpoint concepts. Primary studies were selected for close discussion when they informed DNA localization, sensor perturbation, cellular lineage, or vascular/plaque endpoints; reviews provided mechanistic context. Table 1 presents illustrative studies, while Supplementary Table S2 records the primary studies considered for this focused assessment; neither is intended as an exhaustive field inventory. The L/N/C/E scheme is an author-developed heuristic framework for calibrating evidence-to-claim strength, rather than a validated risk-of-bias tool or a formal systematic evidence-grading instrument. The focused assessment was updated through the final search date. Initial L/N/C/E coding was performed by the lead author study-by-study against the written criteria in Section 2.3. Evidence from different experimental systems or lineages was not combined to create a complete chain. Because this was a focused narrative review, independent double-coding, formal adjudication, and an inter-rater reliability statistic were not performed; ambiguous cases were assigned the lower score, and matched rescue was recorded as an additional confidence feature rather than a requirement for every causal claim.

**TABLE 1:** Representative primary studies defining the evidence boundary for cytosolic DNA sensing in atherosclerosis.

Study/model	L	N†	C	E	Maximum claim/key gap
(Pham et al., 2021) *Apoe* ^−/−^; systemic/BM	0	2†	1	1	Hematopoietic STING-node-dependent lesion inflammation; DNA source, cGAS, and producing cell unresolved
(Dong et al., 2024) OSS ECs + disturbed flow	2	2†	2	2	Cellular mtDNA–cGAMP evidence alongside *in vivo* endothelial STING-node dependence; no EC *Cgas* loss/rescue in the same model
(Sakai et al., 2023) VSMC culture	1	1	1	0	DNA-damage-associated STING-node program; no cGAMP, lineage *in vivo*, or structural endpoint
(An et al., 2023) EC culture	1	1	1	0	Mitochondrial injury-associated STING-node pyroptosis; direct engagement and vascular relevance untested
(Zheng et al., 2025) LyzM-Cre *Mettl4*; *Apoe* ^−/−^	2	0	2	2	Myeloid METTL4-mtDNA and plaque phenotype; cGAS binding, cGAMP, and STING causality untested
(Fidler et al., 2021)*Jak2*V617F BM context	0	2	1	1	CHIP-associated AIM2 inflammation; ligand and cGAS–STING relation unresolved
(Paulin et al., 2018) Advanced-plaque model	1	2	0	2	AIM2 perturbation with cap/collagen features; active DNA, lineage, and rescue unresolved
(Pan et al., 2018) *Apoe* ^−/−^; AIM2 gain/loss	0	2	0	1	AIM2-associated lesion progression and VSMC migration; ligand, output, and VSMC *in vivo* causality unresolved
(Hsu et al., 2023) T1D; *Ldlr* ^−/−^ BM chimeras	0	2	1	2	Hematopoietic AIM2/NLRP3 lesion growth; necrotic core not assigned to AIM2–GSDMD.
(Wang et al., 2024)oxLDL macrophages; TUDCA	0	2	1	1	AIM2-associated ABCA1/efflux and foam cells; systemic TUDCA is not macrophage-AIM2 proof
(Cao et al., 2024) Post-stroke NET-DNA	1	1	0	2	Acute NET-DNA/AIM2 event amplification; sensing-cell entry and chronic relevance unresolved

L/N/C/E definitions and the same-model coherence rule are provided in Section 2.3 and Supplementary Table S3. Table 1 applies these descriptors to representative studies. Among the representative studies summarized here, none fulfills the complete preclinical benchmark. †N2-STING, denotes direct STING-node dependence without same-cell cGAS-to-STING, linkage. cGAMP, evidence is tiered as cGAMP, detected, cGAS-dependent same-cell signaling, or genetic rescue/epistasis; detection alone does not identify the producing cell. Dong et al. is therefore interpreted as cellular mtDNA–cGAMP, evidence alongside *in vivo* endothelial STING-node dependence, not as a complete endothelial cell-autonomous cGAS–STING, chain. These axis scores should not be read as a complete causal chain when ligand evidence and *in vivo* node dependence arise from different experimental layers. Abbreviations: EC, endothelial cell; OSS, oscillatory shear stress; BM, bone marrow; PA, palmitic acid; CKD, chronic kidney disease. For Zheng et al., N = 0 reflects the absence of direct or indirect cGAS/STING, node perturbation or node-specific causal evidence; mtDNA-associated inflammatory and plaque phenotypes do not by themselves support an N score. FFA, free fatty acid; hVSMC, human vascular smooth muscle cell; CHIP, clonal hematopoiesis of indeterminate potential; NET-DNA, neutrophil extracellular trap-derived DNA.

This review focuses on cytosolic self-DNA sensing by cGAS and AIM2. TLR9 principally samples DNA in endosomal rather than cytosolic compartments, and NLRP3 is activated by diverse danger signals rather than DNA directly. IFI16, DDX41, and related DNA-responsive pathways are not included in the core framework because direct *in vivo* causal evidence in atherosclerosis remains insufficient. These pathways are therefore treated as competing or cooperating context, rather than as core components of the cytosolic-DNA evidence framework.

Disease context is interpreted in three scope layers. Core atherosclerosis evidence comprises *Apoe* ^−/−^ or *Ldlr* ^−/−^ models and human plaques with defined plaque endpoints. Atherosclerosis-modifier contexts, including chronic kidney disease (CKD), diabetes, clonal hematopoiesis, aging, smoking, radiation, and acute post-stroke states, can support a mechanism within that background but should not be generalized to baseline atherosclerosis. Vascular extrapolation evidence, including endothelial culture, vascular injury, reendothelialization, and non-plaque senescence models, can resolve cellular mechanisms but cannot establish plaque causality. Table 2 labels these layers separately from L-N-C-E causal-evidence scores.

**TABLE 2:** Disease-context map for candidate self-DNA sensing routes in atherosclerosis.

Context/trigger	DNA source/status	Cell/node	Output/boundary
Core plaque model: OSS flow	mtDNA in OSS EC cytosol; plaque-EC localization unresolved	Endothelium; STING node, with cellular mtDNA–cGAMP evidence	Early dysfunction; STING node, no EC *Cgas* loss/rescue (Dong et al., 2024)
Vascular model:PA/FFA	mtDNA in PA-exposed ECs; plaque-EC localization unresolved	Endothelium; cGAS-dependent STING/IRF3	Adhesion/EndMT in culture; no EC *Cgas* *in vivo* test (Liu et al., 2022a; Mao et al., 2017)
Plaque/vascular model:oxLDL	mtDNA inferred; METTL4-mtDNA 6 mA observed; cGAS binding/cGAMP unmeasured	Macrophages/EC; STING association	Inflammation/foam cells; no macrophage cGAS causality (An et al., 2023; Natarajan et al., 2024). METTL4 effect is not a cGAS–STING chain (Zheng et al., 2025)
Vascular model:oxLDL foam cells	AIM2 ligand and cytosolic DNA not established	Macrophages; PERK–AIM2; ABCA1	Lower efflux/greater lipid storage; TUDCA is not AIM2-specific (Wang et al., 2024)
Modifier: CKD	DNA–cGAS colocalization in fibrous-cap VSMCs; mtDNA identity shown in CKD-serum-exposed hVSMCs	Fibrous-cap VSMCs; cGAS-associated and STING-node signaling	IFN-I, senescence, and switching; structural features were reported in the CKD-associated model, but VSMC-specific cap/collagen causality was not tested (Bi et al., 2021)
Modifier: diabetes	DNA identity unresolved in plaque cells	BM NLRP3/AIM2; GSDMD	Lesion growth; necrotic core not assigned to AIM2–GSDMD (Hsu et al., 2023)
Modifier/vascular model:aging	Micronuclei/chromatin fragments in injury models; plaque localization unresolved	VSMCs; DNA-stress-associated STING-node signaling	SASP/senescence; no plaque structural causality (Uryga et al., 2021; Zheng et al., 2024)
Modifier *JAK2*V617F CHIP	Replication-stress DNA; species/localization undefined	Myeloid/BM; AIM2	Caspase-1/IL-1β inflammation; cGAS–AIM2 ordering unresolved (Fidler et al., 2021)
Acute event:post-stroke	NET-DNA/cfDNA; plaque-cell entry unresolved	Plaque myeloid cells; AIM2 inflammasome	Acute inflammation/thrombosis/recurrence; not chronic baseline risk (Cao et al., 2024)

Human evidence note. Tissue detection supports plaque-compartment relevance but not the producing cell; cell-type association supports localization but not intracellular DNA, sensing; and a same-cell mechanism requires DNA-cGAS, engagement, cGAMP, and activated STING, signaling in one annotated cell. This level has not yet been established in human atherosclerotic plaque.

Evidence-level definitions are provided in Table 1. Table 2 applies these definitions across disease contexts: its stage labels use the minimum endpoint criteria defined in Section 2, whereas DNA-source evidence labels assess DNA, identity and localization independently of the whole-study evidence level in the final column. Abbreviations: EC, endothelial cell; OSS, oscillatory shear stress; PA, palmitic acid; FFA, free fatty acid; CKD, chronic kidney disease. BM, bone marrow; hVSMC, human vascular smooth muscle cell; CHIP, clonal hematopoiesis of indeterminate potential; NET-DNA, neutrophil extracellular trap-derived DNA.

## 2 Evidence rules: a stepwise route from DNA source to claim

Reading guide. The framework is applied in a fixed sequence: first identify a defined and accessible DNA source, then establish the relevant sensor node and cellular lineage, and finally judge the claim against a disease-stage-matched endpoint. Study-level qualifications are concentrated in Tables 1–3 and Supplementary Tables S1–S3 so that the following sections can emphasize the principal biological message.

**TABLE 3:** Decisive experiments and permitted therapeutic inferences across cell compartments and disease contexts.

Cell/setting	Current highest evidence	Critical gap	Decisive experiment	Permitted therapeutic inference
Endothelium	Endothelial STING-node dependence in disturbed-flow models	Endothelial *Cgas* loss/rescue; same-cell DNA/cGAMP evidence	EC-specific *Cgas* deletion ± cGAMP rescue; DNA–cGAS/cGAMP assays; barrier, adhesion, or vasomotor endpoints	Early endothelial dysfunction
Macrophage/hematopoietic	Hematopoietic/STING-node dependence	Plaque-macrophage DNA source and cGAS requirement	Macrophage-specific *Cgas*/*Tmem173* epistasis ± rescue; DNA–cGAS/cGAMP assays; efferocytosis and necrotic-core endpoints	Lesion-progression hypothesis
VSMC	DNA-stress evidence with cGAS-associated and STING-node signaling	VSMC-intrinsic structural causality	*Myh11*-restricted *Cgas*/*Tmem173* perturbation ± rescue; fibrous-cap, collagen, and necrotic-core endpoints	Structural-risk hypothesis
AIM2	Context-specific AIM2-node dependence	DNA entry and effector lineage	Lineage-restricted AIM2 perturbation ± rescue; NET-DNA or CHIP model with a matched acute-event or plaque endpoint	CHIP/NET-DNA-defined settings

Abbreviations: EC, endothelial cell; hVSMC, human vascular smooth muscle cell; CHIP, clonal hematopoiesis of indeterminate potential; NET-DNA, neutrophil extracellular trap-derived DNA.

The matrix translates the evidence framework into experiment-specific, stage-matched therapeutic claims; it is not a cross-cell efficacy ranking.

### 2.1 Step 1: DNA identity, compartment escape, and intracellular localization

#### 2.1.1 Source attribution

Micronuclear DNA, cytosolic chromatin fragments, telomeric fragments, and mitochondrial DNA (mtDNA) are plausible ligands for cytosolic sensors(Gluck et al., 2017; Li and Chen, 2018). Robust source attribution requires identification of the DNA species and its intracellular localization, ideally together with sensor engagement or rescue. Oxidative DNA damage, total cell-free DNA (cfDNA), or mitochondrial dysfunction alone describe upstream context; they do not directly establish cytosolic sensing. mtDNA is especially susceptible to oxidative injury and may reach the cytosol when mitochondrial quality control fails. Its physical presence in the relevant cell compartment, however, should be demonstrated rather than inferred (Ding et al., 2024; Li et al., 2023b; Zheng et al., 2025). To prevent source attribution from being conflated with whole-study causality, Table 2 labels DNA-source evidence independently as directly observed in the specified model, supported but not localized in plaque cells, inferred candidate source, or not established.

#### 2.1.2 Mechanistic boundary

Productive cGAS–STING activation in vascular cells requires more than mitochondrial dysfunction, nuclear DNA damage, or increased total cellular DNA. A credible pathway begins when a defined self-DNA species becomes mislocalized or otherwise available for productive cGAS engagement. mtDNA may enter the cytosol after mitochondrial outer-membrane permeabilization, VDAC oligomerization, permeability-transition-associated injury, or incomplete permeabilization of stressed mitochondria. Nuclear DNA may become accessible through micronuclear-envelope instability, nuclear-envelope herniation, or cytosolic chromatin fragments generated during persistent DNA damage and senescence. Oxidized DNA is a biochemical state of mitochondrial or nuclear DNA, not a distinct source. Mitochondrial stress or DNA damage alone does not establish cGAS activation. The relevant DNA species must be shown outside its native compartment and available for cGAS engagement (Ding et al., 2024; Gluck et al., 2017; Kim et al., 2023; Li and Chen, 2018).

### 2.2 Step 2: productive cGAS–STING signaling and pathway context

#### 2.2.1 Evidence requirement

Evidence should combine imaging of cytosolic DNA localization and cGAS colocalization with biochemical fractionation, DNA-species-specific quantification, cGAMP measurement, and cGAS perturbation.

cGAMP evidence should be interpreted in three tiers: (i) cGAMP detected, which shows that cGAMP is present in a sample or cell population but does not identify the producing cell; (ii) cGAS-dependent same-cell signaling, which requires that Cgas loss or inhibition in the same defined lineage lowers cGAMP together with STING–TBK1–IRF3 signaling and a matched functional endpoint; and (iii) genetic rescue/epistasis, in which 2′3′-cGAMP or a downstream STING agonist restores signaling after Cgas loss, or an upstream–downstream Cgas–Tmem173 relationship is demonstrated genetically. Only the latter two tiers approach cell-autonomous pathway attribution.

#### 2.2.2 Intercellular-transfer boundary

cGAMP can act as an intercellular immunotransmitter through gap-junction transfer, transporter-mediated uptake, and ABCC1-dependent export; its extracellular availability is constrained by ENPP1-mediated hydrolysis (Ablasser et al., 2013b; Dvorkin et al., 2024; Li et al., 2014; Maltbaek et al., 2022; Ritchie et al., 2022; Ritchie et al., 2019). These mechanisms were defined outside atherosclerosis and require direct validation in the relevant vascular compartment. Thus, unless a defined DNA species, cGAS engagement, and cGAMP are shown in the same cell, STING activation in a neighboring vascular cell should not be interpreted as cell-autonomous cGAS activation.

#### 2.2.3 STING activation step

After cGAMP binding, the endoplasmic-reticulum-resident adaptor STING oligomerises, traffics through the ER-Golgi intermediate compartment and Golgi, and recruits TBK1 to promote IRF3 phosphorylation and type I interferon signaling (Mukai et al., 2016; Ogawa et al., 2018). STING can also engage NF-κB and non-canonical stress programs, including PERK-associated responses in endothelium (Kim et al., 2023; Li et al., 2023b). These outputs may contribute to inflammatory activation, senescence-associated secretory phenotypes, or inflammasome-linked pyroptosis, but each requires its own defining evidence. STING inhibition should therefore be interpreted as evidence of STING-node dependence unless the upstream events have been resolved directly (An et al., 2023; Li et al., 2023b).

#### 2.2.4 Pathway-order boundary

Mechanistic convergence does not establish pathway order. The same accessible DNA pool may engage cGAS and AIM2 in one lesion or in different vascular and immune-cell populations. cGAS-derived cGAMP is not required for AIM2 inflammasome assembly, and AIM2 activity does not establish upstream cGAS–STING activation. Interactions in atherosclerosis may instead be indirect. STING-node-dependent inflammatory signaling, mitochondrial injury, impaired efferocytosis, and cell death can alter DNA availability, whereas AIM2-driven IL-1β/IL-18 release and gasdermin D (GSDMD)-dependent lysis can intensify tissue injury and extracellular-DNA exposure. The available evidence therefore supports context-dependent cross-amplification rather than an obligatory cGAS–STING-to-AIM2 cascade.

#### 2.2.5 Potential negative cross-regulation

Negative cross-regulation is also biologically plausible: in macrophages exposed to cytosolic DNA or Francisella novicida, AIM2–caspase-1-activated GSDMD pores induced K^+^ efflux that suppressed cGAS-dependent cGAMP production and IFN-β output independently of pyroptosis and IL-1/IL-18 release (Banerjee et al., 2018). This AIM2–GSDMD-to-cGAS brake was defined outside atherosclerosis; whether it operates in atheromatous cells or plaques, and how it balances AIM2-associated tissue injury, remains untested. Establishing causal order will require cell-specific epistasis experiments with cGAS, STING, and AIM2 loss-of-function and rescue (Cao et al., 2024; Fernandes-Alnemri et al., 2009; Fidler et al., 2021; Paulin et al., 2018).

#### 2.2.6 Scope boundary

cGAS–STING and AIM2 are not the only DNA- and danger-sensing pathways relevant to atherosclerosis. TLR9 samples DNA in endosomal rather than cytosolic compartments, and its effects appear exposure- and context-sensitive. Damaged mitochondrial DNA was linked to proinflammatory TLR9 signaling in an electronic-cigarette model, whereas genetic TLR9 deficiency exacerbated atherosclerosis in another *Apoe* ^−/−^ model (Koulis et al., 2014; Li et al., 2021). NLRP3 is activated by cholesterol crystals and other cellular stresses, providing a competing inflammasome route with a distinct proximal trigger (Duewell et al., 2010). These pathways may converge on inflammatory outputs, but differ in ligand accessibility, subcellular compartment, and effector machinery. They are considered here as competing or cooperating context, not as equivalent alternatives to the cytosolic DNA-sensing axes examined in detail.

### 2.3 Step 3: evidence tiers and disease-stage-matched endpoints

#### 2.3.1 Stage definitions

Disease stage also requires explicit definition. Initiation-relevant arterial dysfunction requires an atheroprone context together with endothelial activation, altered barrier function, leukocyte recruitment, or vasomotor impairment. Progression-associated inflammation requires lesion and inflammatory-cell outcomes. In animal models, structural-risk claims should rest on morphology-defined features such as cap thinning, collagen loss, necrotic-core expansion, matrix-protease activity, thrombosis, or a rupture-prone phenotype. Lesion area alone should not be equated with human plaque vulnerability (Bentzon et al., 2014; Otsuka et al., 2015; Stary et al., 1995; Tabas and Bornfeldt, 2016). Acute stroke-associated recurrence is a distinct systemic-event context, not a surrogate for chronic baseline structural risk.

#### 2.3.2 Minimum endpoint thresholds

Operational disease-stage definitions. In this Review, an early endothelial dysfunction model requires an atheroprone context plus at least one functional endpoint: barrier permeability, leukocyte adhesion, nitric-oxide bioavailability, or vasomotor relaxation. A lesion-progression model requires a lesion outcome together with an inflammatory-cell, foam-cell, efferocytosis, or necrotic-core-remodeling endpoint. A morphology-defined plaque structural risk model requires at least two structural endpoints among fibrous-cap thickness, collagen content or organization, necrotic-core area, matrix-protease activity, intraplaque hemorrhage, or thrombosis; lesion area alone is insufficient. An acute event-amplification model requires a defined systemic event, its candidate DNA source or sensing axis, and a matched acute plaque, thrombosis, or recurrent vascular-event endpoint. These definitions are minimum evidentiary thresholds, not surrogate definitions of human plaque vulnerability (Bentzon et al., 2014; Cao et al., 2024; Otsuka et al., 2015; Tabas and Bornfeldt, 2016).

Scoring rules. Interventions also differ in what they can establish. Whole-body knockout and systemic pharmacology can nominate a pathway but cannot identify the compartment responsible for benefit. Bone-marrow transfer supports a hematopoietic contribution, not necessarily a macrophage-intrinsic route. Conversely, endothelial-specific deletion localizes the manipulated lineage but may leave DNA species, upstream cGAS dependence, and artery-level physiology unresolved. We therefore apply four author-developed heuristic axes: ligand/source attribution (L), node causality (N), cell-lineage specificity (C), and endpoint relevance (E). Each axis is scored from 0 to 2: 0, not established; 1, indirect, association-level, compartment-level, or culture-restricted support; and 2, directly demonstrated in the stated model. L2 requires a defined DNA species and its intracellular accessibility or localization in the relevant model. C2 requires lineage-restricted perturbation in the specified compartment; lineage-matched rescue is recorded as an additional confidence feature (+R). E2 requires a disease-stage-matched vascular or plaque endpoint meeting the minimum criteria above, including at least two morphology-defined endpoints for structural-risk claims.

Same-model coherence and claim boundaries. For a cGAS–STING claim, N2 additionally requires same-cell cGAS-to-STING linkage supported by cGAMP or an equivalent direct readout. Direct STING-node perturbation without resolved upstream cGAS is recorded as N2-STING, not as complete cGAS–STING causality. A complete preclinical direct causal-chain benchmark requires L2/N2/C2/E2 to be linked within the same disease model and specified lineage; evidence cannot be assembled across companion culture and *in vivo* experiments. This benchmark is a stringent operational standard for a complete mechanism, not a general requirement for every causal claim or a quantitative ranking of biological importance. Human spatial evidence remains a separate translational tier. The resulting L/N/C/E assignments for representative studies are summarized in Table 1.

#### 2.3.3 Transition to cell-specific models

Across experimental vascular contexts, self-DNA sensing is better viewed as a set of disease-stage-specific routes than as a single pathway. In oscillatory-shear-stress (OSS)-exposed endothelial cells, disturbed flow is associated with mitochondrial injury and cytoplasmic mtDNA exposure; whether the same event occurs in atherosclerotic plaque endothelium remains unresolved. CKD-associated oxidative stress may promote mtDNA release in VSMCs. In macrophages, lipid stress is linked to mitochondrial remodeling and STING-associated inflammation, although the identity and cytosolic localization of the responsible DNA remain unresolved. By contrast, *JAK2*V617F clonal hematopoiesis is most strongly linked to replication-stress-associated AIM2 activation, and post-stroke NETosis provides an acute systemic source of extracellular DNA that amplifies plaque AIM2 signaling. DNA origin, the route of compartment escape, sensor usage, and pathological output should be assigned separately in each vascular context (Bi et al., 2021; Cao et al., 2024; Dong et al., 2024; Fidler et al., 2021; Natarajan et al., 2024; Uryga et al., 2021).

To navigate the models below, first identify whether a defined endogenous DNA source is demonstrated in the relevant compartment. Next, specify the sensor node and the lineage in which it is perturbed; only then should stage-matched vascular or plaque endpoints determine the maximum claim. The following sections use this sequence to assess endothelial, VSMC, and macrophage/hematopoietic settings, with AIM2 considered as a parallel context-dependent axis.

## 3 Cell- and stage-specific evidence for cGAS–STING

### 3.1 Endothelium

#### 3.1.1 Direct disturbed-flow evidence

In endothelial cells, the most direct evidence supports STING-node-dependent signaling as a contributor to early dysfunction under disturbed flow (Dong et al., 2024). In OSS-exposed endothelial cells, cytosolic dsDNA was detected, mtDNA was identified as the predominant source, cGAS-associated mtDNA and cGAMP were measured, and endothelial *Tmem173* deficiency or knockdown, as well as pharmacologic STING inhibition, reduced senescence, permeability, and lesion-associated outcomes (Dong et al., 2024). This combination is interpreted as cellular mtDNA–cGAMP evidence alongside *in vivo* endothelial STING-node dependence; it does not establish a complete endothelial cell-autonomous cGAS–STING chain without endothelial Cgas loss/rescue and same-model linkage.

#### 3.1.2 Association-level support in lipid stress and aging

Mitochondrial injury and cytosolic-DNA-associated endothelial inflammation in lipid-stress and aging models also track with STING-pathway activation (An et al., 2023; Song et al., 2026; Xie et al., 2023; Zheng et al., 2024).

#### 3.1.3 Endpoint interpretation

Barrier dysfunction, adhesion, and senescence are the relevant endothelial outputs, mediated in a stress-dependent manner by IRF3/IFN-I, NF-κB-, or PERK-associated programs (Dong et al., 2024; Li et al., 2023b; Zheng et al., 2024).

#### 3.1.4 Candidate lipid-stress routes

Free-fatty-acid (FFA)-induced mitochondrial injury activated STING–IRF3 signaling and endothelial inflammation in diet-induced obesity (Mao et al., 2017). Separately, palmitate-exposed endothelial cells provided cellular evidence for cGAS-dependent STING signaling: mitochondrial injury, inflammation, and endothelial-to-mesenchymal transition (EndMT) were attenuated by cGAS, STING, or IRF3 knockdown (Liu et al., 2022a). Neither model establishes plaque-endothelial mtDNA–cGAS localization or endothelial-autonomous *Cgas* requirement in atherosclerosis.

#### 3.1.5 Critical gap and decisive test

A decisive endothelial experiment would combine lineage-restricted *Cgas* or *Tmem173* loss and rescue with intracytoplasmic DNA localization, cGAMP, and flow-conditioned permeability, adhesion, nitric-oxide, or vasomotor endpoints. Cigarette-smoke and radiation models add DNA-damage context but do not identify the dominant endothelial DNA source (Liu et al., 2024; Ueda et al., 2023).

### 3.2 VSMCs

#### 3.2.1 Cellular DNA-stress evidence

In VSMCs, several forms of DNA stress and injury have been described, but their relationship to STING-node signaling is not uniform. In a DNA-double-strand-break model, STING-node-associated proinflammatory activation was implicated in atherosclerosis (Sakai et al., 2023).

#### 3.2.2 Related VSMC stress phenomena

Telomere damage independently promoted VSMC senescence and immune-cell recruitment after vascular injury (Uryga et al., 2021), whereas activated VSMCs released extracellular traps that promoted atherosclerosis (Zhai et al., 2022).

#### 3.2.3 Permitted conclusion

These studies identify DNA damage, senescence, and extracellular-DNA release as related VSMC stress phenomena; they do not, individually or collectively, establish a complete VSMC-intrinsic cGAS–STING causal chain. General VSMC plasticity and structural roles in atherosclerosis are summarized elsewhere (Basatemur et al., 2019). IFN-I, senescence-associated secretory phenotypes, and phenotypic switching are the principal VSMC outputs discussed here, rather than an established cell-death program. This distinction matters because a cellular stress program can be biologically relevant without yet identifying a driver of cap failure or matrix destabilization *in vivo*.

#### 3.2.4 Lineage-related *in vivo* evidence

Independent VSMC-lineage evidence from SM22α-*Trf2*T188A mice links premature VSMC senescence to cytosolic dsDNA and STING-TBK1-IRF3 activation. These mice developed increased atherosclerotic plaque burden and a persistent de-differentiated VSMC program in plaques, while IRF3 silencing restored TGF-β-responsive contractile-gene expression in VSMCs (Kaistha et al., 2025). This study strengthens the link between VSMC DNA damage, STING-associated signaling, and *in vivo* plaque phenotype, but did not test cap thickness, collagen, or necrotic-core endpoints after VSMC-specific *Cgas* or *Tmem173* perturbation.

#### 3.2.5 CKD-specific cellular and plaque evidence

In CKD-associated Apoe^−/−^ atherosclerosis, Bi et al. reported mitochondrial damage, DNA–cGAS colocalization in fibrous-cap VSMCs, and cGAS-associated mtDNA in CKD-serum-exposed hVSMCs, together with p-TBK1 and p-IRF3 activation, an IFN-I-associated senescence and phenotypic-switch program, and morphology-defined structural features including fibrous-cap thinning (Bi et al., 2021).

#### 3.2.6 Permitted inference and boundary

Global *Cgas* or *Tmem173* deficiency improved structural measures, whereas VSMC-specific Tagln-Cre-mediated *Tmem173* deletion supported a VSMC contribution to IFN-I signaling, senescence, and phenotypic switching but was not paired with VSMC-specific *Cgas* perturbation or lineage-matched rescue and structural endpoint testing. This provides lineage-related *in vivo* evidence, but does not establish VSMC-intrinsic cGAS–STING as a necessary driver of structural risk outside the CKD-associated model.

#### 3.2.7 Related modifier evidence

A subsequent CKD-related VSMC study linked DNA damage and a Brd4–Mettl3 regulatory axis to STING-associated vascular calcification (Zhao et al., 2026). This modifier-context study broadens the relevance of VSMC STING signaling but does not provide an atherosclerotic plaque endpoint or a VSMC-specific cGAS–STING causal chain.

#### 3.2.8 Critical gap and decisive test

A decisive VSMC experiment would combine lineage-restricted *Cgas* or *Tmem173* loss and rescue with intraplaque DNA localization, cap thickness, collagen organization, necrotic core, VSMC fate, protease activity, and a defined senescence or phenotypic-switch endpoint.

### 3.3 Macrophages and hematopoietic cells

#### 3.3.1 Hematopoietic STING-node evidence

In macrophage and hematopoietic compartments, STING-node-dependent inflammatory amplification can contribute to lesion progression. Whole-body perturbation, bone-marrow transfer, and systemic pharmacology support STING-node dependence in defined mouse models (Liu et al., 2025; Pham et al., 2021).

#### 3.3.2 Candidate mtDNA-release route

A macrophage GSDMD model linked mitochondrial perforation, mtDNA leakage, STING-IRF3/NF-κB signaling, and lesion burden (Fan et al., 2024).

#### 3.3.3 Relevant endpoint and boundary

STING outputs in this compartment are inflammatory amplification and migration-related programs; efferocytosis and necrotic-core remodeling require direct lesion-level testing. Because GSDMD-mediated mitochondrial perforation can precede mtDNA leakage, pyroptotic machinery should not be placed uniformly downstream of STING.

#### 3.3.4 Unresolved ligand and cGAS requirements

The endogenous DNA source and upstream cGAS requirement remain incompletely defined. Lipid stress, VCAM-1-associated mitochondrial remodeling, mtDNA synthesis, defective efferocytosis, and inflammatory cell death can create a self-DNA-rich plaque environment (Natarajan et al., 2024). However, they do not consistently establish same-cell DNA-cGAS binding in plaque macrophages; cGAMP transfer and non-cGAS STING inputs remain alternatives.

#### 3.3.5 Maximum supported claim

The strongest supported inference is hematopoietic/STING-node dependence, not macrophage-autonomous cGAS–STING causality.

#### 3.3.6 Exploratory cGAS association

An exploratory RNA-seq study in RAW264.7 macrophages reported that pharmacologic cGAS inhibition reduced inflammatory and interferon-associated transcripts (Lu et al., 2021); it did not define an endogenous DNA source, cGAMP production, or a plaque phenotype.

#### 3.3.7 Adjacent myeloid mtDNA evidence

An atherosclerosis-specific myeloid model supports an adjacent mtDNA route. METTL4-mediated mtDNA N6-methyldeoxyadenosine (6 mA) impaired complex V activity, increased cytoplasmic mtDNA and inflammasome output, and, after LyzM-Cre-mediated *Mettl4* deletion, reduced lesion area, lipid deposition, necrotic core, and plaque macrophages while increasing collagen, cap thickness, and smooth-muscle-cell content (Zheng et al., 2025). It does not show mtDNA-cGAS binding, cGAMP, or STING activation.

#### 3.3.8 Mechanistic extrapolation

In THP-1 cells, TDP-43-associated mitochondrial permeabilization induced cytosolic mtDNA, cell-autonomous cGAS–STING signaling, cGAMP, and NF-κB/type I interferon outputs; inhibition of this cell-autonomous cGAS–STING signaling attenuated inflammation (Yu et al., 2020). This ALS model supports the plausibility of mtDNA release but does not identify the endogenous DNA source in plaque macrophages.

#### 3.3.9 Critical gap and decisive test

A decisive macrophage experiment would combine *Cgas*, *Tmem173,* and AIM2 epistasis or rescue with same-compartment DNA localization and efferocytosis, necrotic-core, matrix, and thrombosis endpoints.

Figure 1 provides a visual synthesis of these cell- and stage-specific routes; detailed evidence boundaries remain in Tables 1–4 and the corresponding supplementary tables.

**FIGURE 1:**
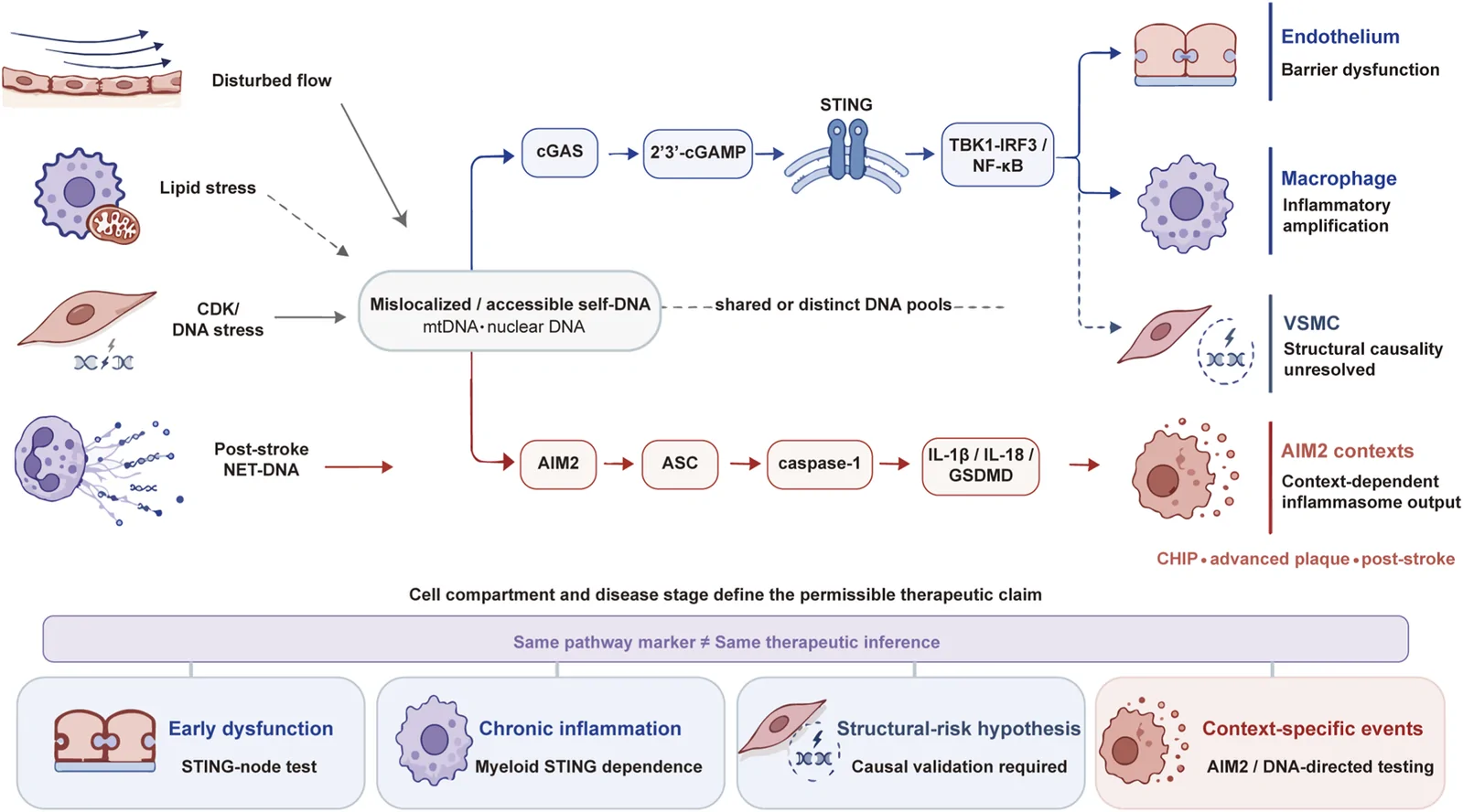
Cell- and stage-specific models of cytosolic self-DNA sensing in atherosclerosis. Disease-context inputs (disturbed flow, lipid stress, CKD-associated or genotoxic stress, and post-stroke NET-DNA) are linked to candidate DNA compartments and cell-specific outputs. Mislocalized mtDNA or nuclear DNA may engage cGAS, generating 2′3′-cGAMP and activating the ER-resident signaling adaptor STING, TBK1–IRF3, and NF-κB. AIM2–ASC–caspase-1 provides a parallel, context-dependent route to IL-1β, IL-18, and GSDMD-associated pyroptosis. Endothelial and macrophage outputs are mapped to early dysfunction and inflammatory amplification; VSMC signaling remains a structural-risk hypothesis. Solid arrows indicate evidence-supported links, dashed arrows incomplete attribution or unresolved causality, and gray arrows unresolved or indirect relationships (including DNA entry, cGAMP transfer, or cross-compartment propagation). Colors denote biological compartments or disease-stage contexts, not evidence strength. cGAMP evidence tiers and the Dong et al. interpretation are defined in Table 1. The schematic is conceptual: solid arrows do not by themselves establish a complete endogenous DNA-to-cGAS-to-STING chain. Abbreviations: mtDNA, mitochondrial DNA; NET-DNA, neutrophil extracellular trap-derived DNA; cGAS, cyclic GMP–AMP synthase; cGAMP, cyclic GMP–AMP; STING, stimulator of interferon genes; TBK1, TANK-binding kinase 1; IRF3, interferon regulatory factor 3; NF-κB, nuclear factor-κB; AIM2, absent in melanoma 2; ASC, apoptosis-associated speck-like protein containing a CARD; GSDMD, gasdermin D; VSMC, vascular smooth muscle cell.

**TABLE 4:** Translational decision matrix for interventions targeting DNA sensing and its upstream or downstream determinants.

Target	Current evidence	Potential advantage	Main constraint	Most suitable setting
cGAS	Cellular mtDNA–cGAMP evidence alongside *in vivo* endothelial STING-node dependence (Dong et al., 2024); human inhibitors (Lama et al., 2019)	Upstream block after L2/N2 evidence	No plaque efficacy; source often unresolved; host-defense risk	L2/N2-defined subgroup
STING	Mouse STING-node dependence; inhibitor tractability/species selectivity; combination-therapy evidence (Haag et al., 2018; Pham et al., 2021; Zhang et al., 2026)	Most tractable *in vivo* node	Human/murine selectivity; infection, surveillance, repair; combination therapy does not establish STING-monotherapy efficacy	Chronic amplification
AIM2	CHIP, advanced-plaque, and post-stroke models (Cao et al., 2024; Fidler et al., 2021; Paulin et al., 2018; Tavallaie et al., 2026)	Context-matched inflammasome intervention	Repair/efferocytosis; bidirectional IL-18 effects	Acute NET-DNA or selected CHIP/advanced plaque
Caspase-1/IL-1β/IL-18	Downstream output; IL-18 paradox in CHIP (Tavallaie et al., 2026)	Output control if sensor identity is unresolved	Host defense, efferocytosis, discordant lesion outcomes	High-output inflammatory states; multi-endpoint testing
DNA clearance	NET-DNA acute-event model (Cao et al., 2024)	Removes ligand before sensing	Delivery; extracellular access; cell-entry uncertainty	NET-DNA-dominant acute event
Upstream lipid/DNA-stress correction	LDL lowering reverses *JAK2*V617F DNA-damage/AIM2 program (Hardaway et al., 2026)	Reduces drivers without broad sensor blockade	Exposure depth, metabolic effects, incomplete source correction	Lipid/DNA-damage modifier context

Table 4 is a translational decision aid, not a cross-target efficacy ranking. Safety domains are required tests, not established adverse effects in every model. Abbreviations: cGAS, cyclic GMP-AMP, synthase; STING, stimulator of interferon genes; AIM2, absent in melanoma 2; IL, interleukin. EC, endothelial cell; OSS, oscillatory shear stress; BM, bone marrow; PA, palmitic acid; FFA, free fatty acid; CKD, chronic kidney disease; hVSMC, human vascular smooth muscle cell; CHIP, clonal hematopoiesis of indeterminate potential; NET-DNA, neutrophil extracellular trap-derived DNA.

## 4 AIM2: a parallel, context-dependent DNA-sensing axis

### 4.1 Activation logic and relationship to cGAS–STING

#### 4.1.1 Activation logic

AIM2 is not established as a downstream extension of cGAS–STING in atherosclerosis. Available data instead support a parallel, context-dependent DNA-sensing axis with a distinct proximal output: ASC recruitment, caspase-1 activation, IL-1β/IL-18 maturation, and potentially gasdermin-D-dependent pyroptosis (Fernandes-Alnemri et al., 2009).

#### 4.1.2 Clonal-hematopoiesis context

AIM2 exacerbates inflammation in JAK2V617F clonal hematopoiesis (Fidler et al., 2021).

#### 4.1.3 Advanced-plaque context

In advanced plaque models, AIM2 perturbation was associated with increased fibrous-cap thickness and lesional collagen content, together with reduced lesional cell death and inflammatory features (Paulin et al., 2018).

#### 4.1.4 Vascular-repair context

Separate AIM2-stimulation studies reported impaired reendothelialization (Lusebrink et al., 2020).

#### 4.1.5 Competing inflammasome route

Cholesterol-crystal-dependent NLRP3 activation is an additional competing inflammasome route (Duewell et al., 2010).

#### 4.1.6 Mechanistically limited direct atherosclerosis evidence

Earlier work provides direct but mechanistically limited atherosclerosis evidence for AIM2. In Apoe^−/−^ mice receiving systemic AIM2 lentiviral gain- or loss-of-function constructs, AIM2 overexpression increased aortic lesion area, lesional smooth-muscle-cell content, and matrix metalloproteinase 2 (MMP2) expression. In cultured VSMCs, oxLDL increased AIM2 expression, and AIM2 perturbation modified VSMC migration and TGF-β/SMAD signaling. However, the responsible DNA species, its cytosolic localization, canonical ASC-caspase-1 output, and VSMC-autonomous *in vivo* causality were not determined (Pan et al., 2018).

### 4.2 Disease-context evidence and therapeutic implications

#### 4.2.1 Diabetes modifier context

Diabetes-accelerated atherosclerosis provides a direct comparison between AIM2 and NLRP3. In streptozotocin- and virus-induced type 1 diabetes models on the Ldlr^−/−^ background, hematopoietic NLRP3 or AIM2 deficiency, including combined deficiency, reduced lesion size. By contrast, the diabetes-associated relative expansion of necrotic core was not prevented by hematopoietic Gsdmd deficiency or by hematopoietic NLRP3/AIM2 deficiency in advanced lesions. These findings support dual hematopoietic inflammasome contributions to lesion growth, but do not identify AIM2–GSDMD as the unique executor of necrosis or pyroptosis (Hsu et al., 2023).

#### 4.2.2 Foam-cell context

Macrophage foam-cell formation represents a further AIM2 context. In oxLDL-exposed macrophages, AIM2 silencing increased ABCA1 abundance and cholesterol efflux capacity, reduced IL-1β/GSDMD-N signals and lipid storage, whereas AIM2 overexpression opposed the ABCA1-restoring effect of tauroursodeoxycholic acid (TUDCA). In *Apoe* ^−/−^ mice, TUDCA reduced plaque area while suppressing the PERK/eIF2α/ATF4-AIM2 program. These data support an AIM2-associated ABCA1/cholesterol-efflux mechanism in macrophages, but the systemic, pleiotropic TUDCA intervention does not establish macrophage-autonomous AIM2 inhibition as the cause of plaque benefit (Wang et al., 2024).

#### 4.2.3 Acute-event context

Among acute event-associated models currently available, post-stroke NET-DNA/AIM2 signaling provides the strongest link to cap, matrix, thrombosis, and recurrent-event phenotypes (Cao et al., 2024). Rapid neutrophil NETosis is the best documented source of the post-stroke cfDNA signal. The route by which NET-DNA becomes cytosol-accessible to plaque sensor cells remains unresolved. This evidence supports a context-restricted inflammatory amplification mechanism; it does not show that AIM2 is a general driver of chronic baseline structural risk.

#### 4.2.4 Unresolved causal ordering

Three models merit direct testing: a shared DNA pool that activates cGAS and AIM2 in 1 cell; spatially segregated signaling in different plaque populations; and indirect cross-amplification through cytokines, mitochondrial injury, impaired efferocytosis, cell death, and DNA release. Only co-registered DNA localization, lineage-restricted single, double, or triple *Cgas*, *Tmem173*, and AIM2 perturbations, and matched rescue can resolve their causal ordering.

#### 4.2.5 Neutrophil source versus sensor-cell boundary

The evidence for different immune-cell populations is asymmetric. In the post-stroke model, neutrophils are best established as the source of NET-DNA that raises circulating cfDNA and amplifies AIM2 signaling in plaques, rather than as the principal cGAS or AIM2 effector cell within the lesion (Cao et al., 2024).

#### 4.2.6 T-cell association boundary

STING expression has also been reported in plaque T-cell-rich regions, but current human data provide an expression association rather than a T-cell-specific DNA-sensing mechanism, lineage-restricted perturbation, or defined pathological output (Pallares et al., 2022).

#### 4.2.7 Hematopoietic boundary

Bone-marrow-transfer and systemic studies support a broader hematopoietic contribution to STING-node-dependent atherogenesis, but do not assign this effect to T cells, neutrophils, or another discrete immune lineage (Liu et al., 2025; Pham et al., 2021).

#### 4.2.8 Modifier-context extension

A newly published PPARα-deficient pathogenic Th17-cell model linked mitochondrial perturbation and mtDNA leakage to STING-node–ERK activation; genetic STING ablation reduced pathogenic Th17-cell expansion and improved macrophage efferocytosis (Ren et al., 2026). This modifier-context evidence broadens the immune-cell map but does not establish a plaque-cell-autonomous mechanism in unselected human atherosclerosis.

## 5 Disease-stage mapping: from DNA sensing to vascular and plaque endpoints

Table 2 first distinguishes core atherosclerosis evidence, modifier contexts, and vascular extrapolation; its DNA-source and endpoint columns then specify what each context can support. Early endothelial dysfunction requires barrier, adhesion, nitric-oxide, or vasomotor measures, whereas lesion-progression models require lesion burden together with inflammatory, foam-cell, efferocytosis, or remodeling readouts. Structural-risk models require at least two morphology-defined endpoints. NET-DNA/AIM2 evidence should be interpreted against matched acute-event endpoints, not chronic baseline plaque risk (Cao et al., 2024; Dong et al., 2024; Fidler et al., 2021; Paulin et al., 2018). Accordingly, a shared pathway marker supports different claims when the cellular compartment, disease stage, or endpoint changes.

## 6 Human evidence and a translational validation roadmap

### 6.1 What human studies currently establish

Human studies establish plaque-compartment relevance and, in selected regions, cell-type-associated STING expression, but not a cell-autonomous endogenous DNA-to-cGAS-to-2′3′-cGAMP-to-STING mechanism. In carotid endarterectomy specimens, Pham et al. detected STING by immunohistochemistry, immunoblotting, and qPCR, and detected 2′3′-cGAMP by LC-MS/MS in five plaques but not in four macroscopically normal carotid arteries (Pham et al., 2021). These tissue-level measurements establish coexistence, not cell-specific cGAS activity. Independently, Pallarés et al. assessed 19 carotid endarterectomy specimens by STING immunohistochemistry on serial sections: staining was heterogeneous, present in endothelial cells, and enriched in macrophage- and T-cell-rich regions, particularly near cell debris and hemorrhagic foci (Pallares et al., 2022). This spatial association does not identify the producing cell, establish local 2′3′-cGAMP synthesis, or demonstrate intracellular DNA sensing.

Single-cell and spatial plaque studies further define endothelial, immune-cell, and VSMC states and associate local programs with fibrous-cap composition, inflammation, or rupture-associated regions (Bleckwehl et al., 2025; Depuydt et al., 2020; Sun et al., 2023). These approaches strengthen spatial association, but transcript abundance cannot establish cGAS activity, cGAMP abundance, or STING protein activation.

### 6.2 What a decisive human study should test

A decisive human study should use carotid endarterectomy specimens to distinguish mitochondrial from nuclear DNA, test DNA-cGAS proximity or binding, quantify cGAMP in microdissected plaque regions, and assess STING, TBK1, and IRF3 activation in the same annotated cells. Matched multiregion histology, plaque microdissection, spatial profiling, and paired plasma cfDNA/mtDNA can distinguish local vascular-wall signaling from systemic tissue injury. Plaque-explant perturbation with cGAS or STING inhibitors, ideally with a rescue condition, would move the evidence from association toward mechanism.

Patient material should match the proposed mechanism. Carotid endarterectomy cohorts are best suited to cell-specific plaque mechanisms; coronary imaging cohorts can test associations between circulating markers, plaque features, and events but not cellular causality; and acute stroke cohorts are suited to the NET-DNA/AIM2 event-amplification hypothesis (Cao et al., 2024). The principal cell- and stage-matched experiments and their permitted therapeutic inferences are summarized in Table 3.

## 7 Pharmacological feasibility is a separate evidentiary gate

### 7.1 Target tractability, delivery, and safety

In VSMCs, macrophages, and other plaque or hematopoietic cells, disease context may set the threshold at which DNA-sensing pathways become therapeutically relevant. Chronic kidney disease, biological aging, cigarette-smoke exposure, and low-dose radiation are associated with DNA damage, mitochondrial stress, or inflammatory cell death. JAK2V617F clonal hematopoiesis can remodel myeloid inflammatory competence (Bi et al., 2021; Fidler et al., 2021; Liu et al., 2024; Ueda et al., 2023; Uryga et al., 2021; Zheng et al., 2024). These settings may change the relative importance of STING or AIM2 through different combinations of DNA release, mitochondrial quality control, cell death, and immune remodeling. They are candidate context modifiers for mechanistic and clinical stratification, not evidence for a shared upstream pathway or a basis for biomarker-guided treatment before prospective validation.

Target tractability, delivery, and safety should be assessed separately from pathway readouts. Key domains include human-versus-murine inhibitor selectivity, plaque and cell-specific exposure, infection susceptibility, tumor immune surveillance, endothelial repair/reendothelialization, macrophage efferocytosis, and tissue repair. These considerations are particularly important when a pathway node has broader innate-immune functions or when a downstream cytokine improves one structural feature while worsening lesion growth or necrosis (Lusebrink et al., 2020; Pham et al., 2021; Tavallaie et al., 2026).

Species selectivity is directly relevant to STING-directed development: DMXAA and CMA activated murine but not human STING, whereas rat and human STING showed more similar pharmacological profiles (Zhang et al., 2015). Activity should therefore be established in human STING variants and relevant human plaque cells before efficacy is extrapolated from murine studies. Tissue-repair effects are also context dependent: in a murine skin-injury model, *Tmem173* deficiency delayed wound closure through impaired myeloid/macrophage trafficking (Chen et al., 2025a). This does not predict plaque repair, but supports retaining endothelial repair, efferocytosis, and tissue-repair endpoints in chronic-inhibition studies. Broader host-defense and tumor-immune-surveillance concerns remain pharmacological risks to evaluate, rather than established adverse effects in atherosclerosis (Decout et al., 2021; Haag et al., 2018).

Chemical tractability at cGAS is also supported outside atherosclerosis: human-cGAS-specific lead compounds occupied the catalytic nucleotide-binding pocket and suppressed DNA-triggered cytokine output in primary human macrophages, with the most selective leads showing no substantial off-target activity across tested innate sensors (Lama et al., 2019). This establishes a pharmacological starting point, but not plaque exposure, *in vivo* efficacy, or cGAS requirement in atherosclerosis.

### 7.2 Context-specific pharmacological evidence

In murine atherosclerosis, the strongest *in vivo* pharmacological proof-of-concept concerns STING. Systemic C-176 reduced atherosclerotic burden and inflammatory activation, whereas bone-marrow STING expression promoted atherogenesis (Pham et al., 2021). These findings support a tractable hematopoietic STING node, but not endogenous DNA identity, cGAS requirement, or a macrophage-selective mechanism. A 2026 nano-immunotherapy combining cholesterol-crystal dissolution with C-176 reduced plaque pathology and restored endothelial barrier features (Zhang et al., 2026); because it targets both crystal burden and STING, it cannot establish STING-inhibitor monotherapy efficacy. Species selectivity, plaque exposure, and long-term safety require direct assessment (Haag et al., 2018).

Natural-product studies with reduced STING-pathway readouts are best treated as pleiotropic associations. In high-fat-diet (HFD)-fed Apoe^−/−^ mice, tetrandrine reduced lesion burden and macrophage STING/TBK1/NF-κB readouts; in HFD-fed Ldlr^−/−^ mice, aucubin reduced aortic STING expression and atherosclerosis while also affecting miR-181a-5p, glycemia, and liver disease. Although tetrandrine suppressed cGAMP- or oxLDL-associated STING/TBK1 signaling in macrophages and aucubin suppressed mtDNA-induced STING/NF-κB signaling in bone-marrow-derived macrophages, neither study demonstrates direct STING engagement, selectivity, cGAS requirement, or a cell-autonomous mechanism. Kuanxiong Aerosol, another multicomponent preparation, was associated with lower plaque inflammation and reduced CX3CR1-positive macrophage apoptosis in high-fat-diet-fed Apoe^−/−^ mice; reversal by STING-pathway activation links the effect to STING signaling but does not establish direct target engagement or cGAS requirement (Chen et al., 2025b). These agents therefore support STING-linked anti-inflammatory activity but should not be ranked with lineage-restricted genetics, rescue, or selective STING inhibitors (Li et al., 2023a; Liu et al., 2022b).

Endothelial STING inhibition remains a prevention-oriented hypothesis. The most direct evidence comes from OSS models, whereas lipid-stress and aging studies report STING-pathway associations with inflammatory activation, senescence, or barrier dysfunction (Dong et al., 2024; Zheng et al., 2024). Before cGAS inhibitors are positioned alongside STING blockade, endothelial studies should demonstrate plaque exposure, reduced cGAMP, concordance with endothelial *Cgas* loss-of-function, and improvement in vascular or plaque-structural endpoints. FFA-induced mitochondrial injury supports a STING–IRF3 inflammatory route (Mao et al., 2017), whereas palmitate-exposed endothelial cells support a cellular cGAS-dependent STING–IRF3 mechanism with adhesion and EndMT outputs (Liu et al., 2022a). Neither model has yet been linked to endothelial-specific *Cgas* loss/rescue in an atherosclerosis model.

AIM2-directed intervention should be evaluated separately in cell-death-rich advanced plaques and acute NET-DNA-exposed plaque compartments. In advanced plaque models, AIM2 strategies were associated with increased lesional collagen and fibrous-cap thickness and reduced cell-death-related features (Paulin et al., 2018). In acute-event models, NET-DNA clearance or inflammasome-directed intervention reduced post-stroke inflammation and recurrent vascular events (Cao et al., 2024). These results support context-specific hypotheses, not long-term systemic AIM2 inhibition for chronic atherosclerosis. AIM2 activation can impair reendothelialization, but this activation model does not substitute for a selective therapeutic-inhibition study (Lusebrink et al., 2020).

The *Jak2*V617F clonal-hematopoiesis model illustrates why a downstream cytokine cannot be evaluated through one structural readout. IL-18 antibody treatment increased plaque collagen content and fibrous-cap thickness, yet increased early lesion area and the frequency of advanced lesions with large necrotic cores. It also reduced plaque AIM2 and cleaved GSDMD, while increasing apoptotic macrophage features and impairing efferocytosis in association with reduced IFN-γ signaling. Thus, lower IL-18 or inflammasome markers can coexist with worse lesion progression and necrosis. Therapeutic assessment should jointly measure lesion burden, necrotic core, efferocytosis, cap/matrix features, and host-defense or tissue-repair safety rather than infer net benefit from collagen or cap thickness alone (Tavallaie et al., 2026).

An alternative is to correct the upstream lipid milieu rather than directly inhibit a DNA-sensing node. In *Ldlr* ^−/−^ mice reconstituted with *Jak2*V617F/WT bone marrow, marked LDL lowering after established disease halted further lesion progression, reduced macrophage burden, and increased lesional collagen. This intervention also reversed the increased DNA-damage response, AIM2 inflammasome activation, and proliferation in *Jak2*V617F macrophages, while promoting TREM2/c-Myc macrophage features associated with resolution. Comparison with moderate LDL lowering indicates that treatment depth and disease stage matter. These data support upstream lipid-stress correction as a testable route to suppress the DNA-damage/AIM2 program; they do not establish that direct cGAS, STING, or AIM2 inhibition is ineffective or unsafe, nor do they establish the particular cholesterol-lowering regimen as a human therapeutic solution (Hardaway et al., 2026).

### 7.3 Therapeutic decision rule

Therapeutic decision rule: test STING inhibition when only STING-node dependence is established; consider cGAS inhibition after same-cell DNA-to-cGAS-to-cGAMP evidence; and compare DNA clearance with AIM2/inflammasome blockade when NET-DNA or acute cell death is dominant. Structural-risk claims require cap, collagen, matrix-turnover, and safety endpoints (Decout et al., 2021). This target-selection sequence is summarized in the translational decision matrix in Table 4.

## 8 Discussion and conclusion

### 8.1 Limitations of the evidence framework

This focused narrative review was not a systematic review and considered English-language literature identified primarily through PubMed and Web of Science, supplemented by cited-reference screening. The L/N/C/E scheme is an author-developed heuristic rather than a validated risk-of-bias or certainty-of-evidence instrument; no formal risk-of-bias assessment or certainty rating was performed. Heterogeneity across cellular systems, genetic and pharmacological models, modifier contexts, disease stages, and endpoint definitions may influence individual assignments and their transportability. The framework should therefore calibrate the scope of inference, not be interpreted as a quantitative ranking of biological importance or as a substitute for systematic evidence synthesis. Because this literature is rapidly evolving, subsequent studies may change individual L/N/C/E assignments.

### 8.2 Conclusion

Established: within the studies considered here, STING-node dependence is supported in selected endothelial and hematopoietic inflammatory models, and AIM2 perturbation is associated with context-specific outputs in clonal hematopoiesis, advanced-plaque models, and post-stroke NET-DNA states. Incomplete: human evidence remains predominantly tissue- or cell-type-associated, while VSMC DNA-stress/STING signaling lacks proof of VSMC-intrinsic structural causality. Hypothesis: therapeutic effects must therefore be tested with cell- and stage-matched endpoints rather than inferred from pathway markers alone.

Not established: the dominant endogenous DNA species in human plaques, lineage-specific *in vivo* cGAS requirement, and the causal ordering of cGAS–STING, AIM2, and NLRP3 remain unresolved; human evidence is currently stronger for tissue or cell-type association than for intracellular mechanism.

Decisive experiments should co-register DNA localization and sensor engagement in a specified lineage and link lineage-restricted perturbation, ideally with rescue, to a matched vascular or plaque endpoint.
