# Supplementary material for: An evidence-to-claim framework for cGAS–STING and AIM2 in atherosclerosis
Source: Front Cell Dev Biol. 2026 Oct 22;14:1999070. doi: 10.3389/fcell.2026.1999070 (PMC13736093; doi:10.3389/fcell.2026.1999070)
Supplement: Supplementary file 1 [file Table1.docx]

**Supplementary Materials**

**Table S1. Decisive next experiments and falsifiable predictions for the focused evidence map.**

**A. Decisive next experiments**

| **Model / study** | **Decisive next experiment** |
| --- | --- |
| Disturbed-flow endothelium (Dong et al., 2024) | Endothelial *Cgas*/*Tmem173* loss and rescue with same-cell mtDNA-cGAS-cGAMP measurement and permeability, adhesion, nitric-oxide, or vasomotor endpoints. |
| Lipid-stress endothelium (Liu et al., 2022a; Mao et al., 2017) | Test endothelial *Cgas* dependence in an atherosclerosis model and link mtDNA localization to EndMT, adhesion, and vascular-function outcomes. |
| Macrophage/ hematopoietic STING (Fan et al., 2024; Pham et al., 2021) | Use macrophage-restricted *Cgas*/*Tmem173* epistasis with DNA localization, cGAMP, efferocytosis, necrotic-core, and matrix endpoints. |
| METTL4-modified mtDNA (Zheng et al., 2025) | Determine whether modified mtDNA binds cGAS, generates cGAMP, and activates STING in plaque macrophages; combine with macrophage-specific rescue. |
| CKD-associated VSMC program (Bi et al., 2021) | Use VSMC-restricted *Cgas* or *Tmem173* loss/rescue with intraplaque DNA localization, cap thickness, collagen organization, necrotic core, VSMC fate, and protease activity. |
| VSMC DNA-damage culture (Sakai et al., 2023) | Move from siRNA/cellular phenotypes to lineage-restricted in vivo perturbation with matched structural plaque endpoints. |
| AIM2 in clonal hematopoiesis (Fidler et al., 2021; Hardaway et al., 2026; Tavallaie et al., 2026) | Apply lineage-restricted Aim2 rescue and compare direct AIM2 blockade with upstream lipid lowering while measuring lesion burden, necrosis, efferocytosis, cap/matrix features, and host defense. |
| Advanced-plaque/foam-cell AIM2 (Pan et al., 2018; Paulin et al., 2018; Wang et al., 2024) | Identify the DNA compartment and sensing lineage, then test Aim2 perturbation with ABCA1/efflux, cell death, and multiple structural endpoints. |
| Post-stroke NET-DNA/AIM2 (Cao et al., 2024) | Localize NET-DNA entry into plaque sensor cells and perform lineage-specific AIM2/cGAS/STING epistasis with acute recurrence, thrombosis, cap, and matrix outcomes. |
| STING-linked pharmacology (Li et al., 2023; Liu et al., 2022b; Zhang et al., 2026) | Separate direct target engagement from multi-target activity using selective STING inhibition, monotherapy arms, plaque exposure, and lineage-resolved readouts. |

**B. Model-specific predictions and falsifying outcomes**

| **Disease context** | **Prediction / falsifying outcome** |
| --- | --- |
| Disturbed or oscillatory flow | Prediction: endothelial mtDNA-cGAS-cGAMP is required for STING-dependent dysfunction. Falsifier: endothelial *Cgas* loss leaves STING activation and functional endpoints unchanged. |
| Lipid stress / EndMT | Prediction: cytosolic mtDNA and cGAS-STING drive EndMT/adhesion in endothelium. Falsifier: cGAS loss does not alter these outputs despite intact mitochondrial stress. |
| Macrophage lipid stress | Prediction: hematopoietic STING dependence tracks inflammation, defective efferocytosis, or necrotic-core remodeling. Falsifier: macrophage *Tmem173* loss does not alter these lesion endpoints. |
| CKD-associated VSMC stress | Prediction: VSMC *Cgas*/*Tmem173* perturbation jointly changes cap, collagen, and necrotic-core measures. Falsifier: only cellular markers change without at least two structural endpoints. |
| JAK2V617F clonal hematopoiesis | Prediction: AIM2-targeted and upstream lipid-stress interventions have separable effects on inflammation and plaque remodeling. Falsifier: altering AIM2 activity does not change the matched disease phenotype. |
| Post-stroke state | Prediction: NET-DNA clearance or AIM2 blockade preferentially reduces acute plaque inflammation and recurrent events. Falsifier: neither intervention changes these endpoints despite documented NET-DNA elevation. |

Abbreviations: EC, endothelial cell; OSS, oscillatory shear stress; BM, bone marrow; PA, palmitic acid; FFA, free fatty acid; CKD, chronic kidney disease; hVSMC, human vascular smooth muscle cell; CHIP, clonal hematopoiesis of indeterminate potential; NET-DNA, neutrophil extracellular trap-derived DNA.

**Table S2. Primary studies considered in the focused narrative assessment.**

| **Study** | **Model / scope layer** | **Compartment / node** | **Perturbation** | **Critical missing L/N/C/E element** | **Maximum supported claim assessment category** |
| --- | --- | --- | --- | --- | --- |
| (Zheng et al., 2025) | Atherosclerosis | Macrophage; METTL4/mtDNA | LyzM-Cre Mettl4 deletion | mtDNA-cGAS binding; cGAMP/STING; rescue | Maximum claim: node dependence |
| (An et al., 2023) | Endothelial mitochondrial stress | Endothelium; cGAS-STING | RU.521; C-176 | DNA-cGAS/cGAMP; in vivo lineage; vascular function | Assessment: cellular mechanism |
| (Cao et al., 2024) | Post-stroke recurrence | NET-DNA/plaque-myeloid; AIM2 | DNase; inflammasome-axis perturbation | Cytosolic entry; sensing cell; chronic generalizability | Assessment: event-associated mechanism |
| (Fidler et al., 2021) | JAK2V617F clonal hematopoiesis | Myeloid/hematopoietic; AIM2 | Aim2; caspase-1/11; Gsdmd genetics | DNA source/localization; cGAS-STING order; Aim2 rescue | Maximum claim: node dependence |
| (Paulin et al., 2018) | Advanced plaque | Lesion lineage unresolved; AIM2 | Aim2 perturbation | DNA compartment; lineage; rescue; cGAS-STING relation | Maximum claim: node dependence |
| (Bi et al., 2021) | CKD-Apoe^−/−^ | Fibrous-cap VSMC; cGAS-STING | Global *Cgas*/*Tmem173;* VSMC *Tmem173* deletion | VSMC *Cgas*/*Tmem173* rescue; multiple structural endpoints | Maximum claim: node dependence |
| (Dong et al., 2024) | Apoe^−/−^ disturbed flow; OSS | Endothelium; STING | Endothelial *Tmem173* deletion; knockdown/inhibition | Endothelial *Cgas* loss/rescue and same-model linkage of cellular mtDNA-cGAMP with in vivo STING/endpoints | Maximum claim: node dependence |
| (Natarajan et al., 2024) | Inflammatory lipid stress | Plaque macrophage; mtDNA pathway | Vcam1/mtDNA-synthesis perturbation | Cytosolic DNA-cGAS; cGAMP; macrophage rescue | Maximum claim: node dependence |
| (Uryga et al., 2021) | Vessel injury/telomere damage | VSMC | Telomere-damage manipulation | Atherosclerosis testing; cGAS-STING perturbation; plaque endpoints | Assessment: vascular extrapolation |
| (Pham et al., 2021) | Hyperlipidemic Apoe^−/−^ | Whole-body/hematopoietic; STING | Global *Tmem173 d*eletion; C-176; bone marrow | DNA source; cGAS requirement; cell-autonomous origin | Maximum claim: node dependence |
| (Sakai et al., 2023) | DNA-break VSMC culture | VSMC; cGAS-STING | cGAS/STING siRNA | DNA localization; cGAMP; in vivo lineage; structural causality | Assessment: cellular mechanism |
| (Zheng et al., 2024) | Ageing endothelial inflammation | Endothelium; STING | STING inhibition; cilostazol | DNA species; cGAS requirement; atherosclerosis-stage validation | Assessment: vascular extrapolation |
| (Song et al., 2026) | LPS-accelerated atherosclerosis | Endothelium; GSDMD-STING | Endothelial Gsdmd deficiency; rescue | DNA-cGAS interaction; *Cgas*/*Tmem173* epistasis; conventional-AS relevance | Maximum claim: node dependence |
| (Xie et al., 2023) | oxLDL atherosclerosis | Endothelium; GSDME-STING | Gsdme deficiency/knockdown | DNA-cGAS/cGAMP; endothelial epistasis | Maximum claim: node dependence |
| (Liu et al., 2024) | Radiation + HFD | Compartment unresolved; cGAS | cGAS inhibition/perturbation | DNA localization; STING; lineage; rescue | Maximum claim: node dependence |
| (Ueda et al., 2023) | Cigarette-smoke mtDNA damage | Vascular/endothelial context; cGAS-STING | Pathway perturbation | Sensing cell; in vivo chain; matched endpoint | Assessment: vascular extrapolation |
| (Zhai et al., 2022) | VSMC extracellular traps | VSMC DNA-donor context | Extracellular-trap perturbation | Cytosolic uptake; sensor node; lineage test; rescue | Assessment: event-associated mechanism |
| (Liu et al., 2025) | Lupus + Apoe^−/−^ | Whole-body immune; STING | Global *Tmem173* deletion; DNase I | AS DNA source; lineage; cGAS-rescue chain | Assessment: modifier context |
| (Fan et al., 2024) | Atherosclerosis | Macrophage; GSDMD-STING | Gsdmd manipulation | Macrophage *Cgas*/*Tmem173* epistasis; DNA-cGAS; rescue | Maximum claim: node dependence |
| (Lusebrink et al., 2020) | Reendothelialization/AS | Endothelial/lesion; AIM2 | AIM2 stimulation | Inhibition test; DNA compartment; lineage; cGAS-STING relation | Assessment: vascular extrapolation |
| (Pallares et al., 2022) | Human carotid plaque | Endothelial/macrophage/T-cell regions; STING | Tissue/cell-type detection | Same-cell DNA-cGAS-cGAMP-STING; functional validation | Assessment: human translational association |
| (Zhang et al., 2026) | Apoe^−/−^ nano-immunotherapy | Plaque/endothelium/foam-cell context; STING | Crystal dissolution + C-176 | DNA source; cGAS; STING monotherapy; lineage; rescue | Maximum claim: node dependence |
| (Lu et al., 2021) | cGAS inflammation model | Compartment unresolved; cGAS | cGAS inhibition/knockdown | DNA identity; STING; lineage; rescue | Assessment: cellular mechanism |
| (Mao et al., 2017) | Diet-induced obesity | Endothelium; STING-IRF3 | STING/IRF3 pathway intervention | DNA identity/localization; cGAS; AS-stage lineage test | Assessment: vascular extrapolation |
| (Liu et al., 2022a) | Palmitate endothelial culture | Endothelium; cGAS-STING | cGAS/STING/IRF3 knockdown | In vivo lineage; plaque endpoint; rescue | Assessment: cellular mechanism |
| (Pan et al., 2018) | Apoe^−/−^ + VSMC culture | VSMC; AIM2 | Systemic AIM2 gain/loss | DNA ligand/localization; canonical output; VSMC in vivo causality | Maximum claim: node dependence |
| (Hsu et al., 2023) | Diabetes-accelerated Ldlr^−/−^ | Hematopoietic; AIM2/NLRP3 | Nlrp3/Aim2/Gsdmd genetics | DNA source; AIM2-specific execution; lineage-rescue chain | Assessment: modifier context |
| (Wang et al., 2024) | Apoe^−/−^ + foam-cell model | Macrophage; AIM2/ABCA1 | TUDCA; AIM2 manipulation | Direct AIM2 target effect; lineage-specific in vivo mechanism | Assessment: pleiotropic pharmacological association |
| (Tavallaie et al., 2026) | JAK2V617F clonal hematopoiesis | Hematopoietic; IL-18 downstream | IL-18 antibody | Direct AIM2 attribution; cell-specific rescue | Assessment: modifier context |
| (Hardaway et al., 2026) | JAK2V617F regression | Hematopoietic macrophage context; AIM2 | Intensive LDL lowering | Direct pathway perturbation; human translation | Assessment: upstream-correction context |
| (Li et al., 2023) | HFD-Apoe^−/−^ | Macrophage/plaque; STING | Tetrandrine | Direct STING engagement; selectivity; cGAS; lineage; rescue | Assessment: pleiotropic pharmacological association |
| (Liu et al., 2022b) | HFD-Ldlr^−/−^ + steatohepatosis | BMDM/aorta/liver; STING | Aucubin | Direct STING engagement; selectivity; cell autonomy; cGAS; rescue | Assessment: pleiotropic pharmacological association |
| (Chen et al., 2025) | HFD-Apoe^−/−^ | Macrophage/plaque; cGAS-STING | Kuanxiong Aerosol; STING-pathway activation | Direct target engagement; selectivity; cGAS requirement; cell-autonomous genetic mechanism | Assessment: pleiotropic pharmacological association |
| (Zhao et al., 2026) | CKD-related vascular calcification | VSMC; Brd4-Mettl3/STING | Brd4/Mettl3/STING modulation | Plaque endpoint; DNA source; cGAMP; VSMC-specific *Cgas*/*Tmem173* epistasis or rescue | Assessment: modifier-context mechanistic association |
| (Ren et al., 2026) | PPARα-deficient pTh17 cells; Apoe^−/−^ modifier context | CD4+ T cells; mtDNA–cGAS–STING–ERK | Ppara deficiency; STING ablation / PPARα activation | Same-cell DNA localization and cGAMP not established; human causality unresolved | Immune-cell modifier-context evidence; not a general plaque-cell-autonomous mechanism |

**Table S3. Literature approach and heuristic coding schema for the focused narrative assessment.**

| **Selection element** | **Transparent description** |
| --- | --- |
| Last search date | 6 October 2026. |
| Data sources | PubMed and Web of Science, supplemented by backward cited-reference screening. |
| Principal search construct | (atherosclerosis OR vascular inflammation OR carotid plaque) AND (cGAS OR STING OR AIM2 OR cytosolic DNA OR mitochondrial DNA), combined with cell-compartment and endpoint terms. |
| Focused eligibility | English-language primary studies in atherosclerosis or a directly relevant vascular context that informed DNA source/localization, sensor perturbation, cellular compartment, or vascular/morphology-defined endpoints. Reviews were used only for context. |
| Model / scope layer | The Table S2 Model / scope layer column classifies each study as core atherosclerosis evidence, an atherosclerosis-modifier context, or vascular extrapolation evidence. Scope informs transportability; it is not an L/N/C/E descriptor. |
| Heuristic framework | L = ligand/source attribution; N = node causality; C = cell-lineage specificity; E = endpoint relevance. Each axis uses 0–2: 0, not established; 1, indirect, association-level, compartment-level, or culture-restricted support; 2, directly demonstrated in the stated model. L2 requires an identified DNA species plus intracellular accessibility/localization. N2 requires direct node perturbation with a proximal output; for cGAS–STING, N2 also requires same-cell cGAS-to-STING linkage supported by cGAMP or equivalent direct evidence. N2-STING records direct STING-node dependence without upstream cGAS linkage. C2 requires lineage-restricted perturbation; +R records matched rescue. E2 requires a stage-matched vascular/plaque endpoint, with at least two morphology-defined endpoints for structural-risk claims. A complete preclinical chain requires L2/N2/C2/E2 in the same model and specified lineage. |
| Coding procedure | Each primary study was coded study-by-study against the written L/N/C/E criteria. Evidence from different experimental systems or lineages was not combined to create a complete chain. The coding was an author-applied heuristic rather than an independent dual-reviewer exercise; no inter-rater reliability statistic or formal adjudication process was used. When a report was ambiguous, the lower descriptor was assigned. Rescue increased confidence (+R) but was not required for every causal claim. The coding unit was the defined model–lineage–endpoint combination reported in each primary study; unresolved DNA identity, sensor engagement, lineage assignment, or stage-matched endpoint evidence lowered the corresponding axis rather than being inferred from pathway association. |
| Studies selected | Thirty-five primary studies are listed in Table S2. The list is an audit trail for this focused assessment, not an exhaustive inventory of the field. |
| Table 1 selection | Table 1 is a representative subset of Table S2 selected to span endothelial, VSMC, hematopoietic/macrophage, and AIM2 settings and to illustrate different L-N-C-E profiles. |
| Exclusions | General pathway reviews, canonical mechanistic papers, and non-atherosclerotic comparators were not tabulated as disease-context evidence. TLR9 and NLRP3 were retained as scope-defining competing pathways but excluded from the core cytosolic-DNA evidence grading. |
| Review-method boundary | This focused narrative review reports neither PRISMA-style record counts nor dual-reviewer/inter-rater metrics; the tables are a qualitative audit, not a systematic-review flow or exhaustive inventory. |
| Assessment category | The final Table S2 column reports either the maximum supported claim or an assessment category. These labels are not additional L/N/C/E levels. |
